# Fistula in Ano

**Published:** 1841-10

**Authors:** M. L. Linton

**Affiliations:** Springfield Ky


### FISTULA IN ANO. BY M. L. LINTON.

I flatter myself that the present communication, though a very brief one, will prove serviceable, not only to the profession, but to the public in general. Its design is to give notoriety to some important facts in regard to the treatment of *Fistula in Ano*—a disease, the treatment of which is as simple as any other in surgery, when the knife is to be used. But the horror which most persons have of cutting instruments, has very naturally led to the searching out and substituting of various other methods, amongst which is that by the ligature, which will do away with the necessity of the knife in nearly all if not all cases.

I do not say that it is better than the knife, but the truth is, that patients generally prefer it; and the surgeon is bound to regard their prejudices, when it can be done with safety.

We have lately treated two cases in this manner with perfect success.

The first was a negro woman, aged 25 years. She had been afflicted with the disease between six and seven years; and her health was considerably impaired. The fistula was complete. I introduced through the external orifice of the fistulous canal, by means of a slippery elm bougie, one end of a common flaxen cord, which I brought out at the end of the anus. The part to be cut through was thus included in the ligature, which I tied in a manner that I cannot very easily describe. No matter. The twisting of the two ends of the ligature, which should be well waxed, or tying them in what is called the single bow knot, answers as well as any other *can.*

The treatment from this time until a perfect cure was effected—the space of four weeks—consisted in tightening the cord every day or every other day—introducing, occasionally, into the fistulous canal a slippery elm bougie dipped in a solution of nitrate of silver, four grains to the ounce of water, keeping the bowels loose, and allaying irritation by an occasional opiate.

I attended this patient but little as she resided three miles from town—but her case was well and faithfully managed by my friend and pupil, J. R. Hughes, whose intelligence, industry, and zeal, justify hopes of future usefulness.

The second patient was a man upwards of thirty years of age. This case presented itself shortly after the termination of the foregoing.

My colleague, Dr. Polin, introduced a common silk thread, and proceeding in other respects as has already been described, until the cure was nearly effected, when the patient becoming very *impatient* the Doctor brought the knife to the aid of the ligature, though the latter would have done the work in a few more days.

As a general rule about three weeks are sufficient to effect a cure, in almost every case; for in the former of those just detailed, the fistula opened externally three inches from the anus; and in the latter the internal orifice was nearly three inches above the sphincter.

The fistula heals "pari passu" with the progress of the ligature, so that when the part is cut through the cure is completed. The use of the caustic is to destroy the deceased and indurated parietes of the fistulous canal. This may be left off after the first week, or it may be used for a week or so before the ligature is applied. In order that this mode of operating may produce but little pain and irritation, the ligature should be tightened frequently, and but *very little at each time.*

I have already said that I would not assert the superiority of the system over the knife; but it is well known that patients generally prefer it, and will follow a quack as long as they can hear of one who promises to cure without cutting.

The operation by ligature is as old as surgery itself. It is alluded to by Hippocrates—was in frequent use in the time of Celsus, and employed by surgeons in all ages from that to the present—some using wires of the various metals, some horse-hair, and some a common thread; yet this old operation is now being palmed upon the public as something entirely *new and secret*, by a Dr. Bodenhamer, of Paris, Ky. True he cures many of his cases, but then they might be equally well cured without the fatigue of a journey to Paris, and for a fee a great deal less than two hundred dollars. Any country physician or student, with the directions here given, can perform the operation as well as any one else.

I assert that this is substantially the operation of Bodenhamer on the authority of his own patients; and my only apology for making the communication is, that I am a friend to mankind, and a bitter enemy to nostrums and nostrum-mongers.

*Springfield Ky,* 1841.

